# Special Issue: Intensive Care for Critically Ill Neonates: Clinical Diagnosis and Treatment

**DOI:** 10.3390/children10071203

**Published:** 2023-07-11

**Authors:** Stefan Grosek

**Affiliations:** Department of Perinatology, Division of Gynaecology, University Medical Centre Ljubljana, 1000 Ljubljana, Slovenia; stefan.grosek@kclj.si; Tel.: +386-41-684-206

Many physicians and researchers in the recent past have recognized the need to provide care and to study term and preterm infants when sufficient knowledge had not yet been attained, and to research how to approach and care for the most vulnerable children, i.e., sick preterm and term newborns who may or may not develop critical illnesses. Discovering and understanding the physiology and pathophysiology that may occur during foetal life, the transition from intrauterine to extrauterine life and postnatal days, has helped perinatologists, obstetricians, and neonatologists to better follow-up and prepare the mother and foetus before delivery and for birth, and delivery teams to act appropriately and efficiently if the newborn has developed envisaged or unexpected problems. 

Microtechnology and macrotechnology have steadily developed and have been increasingly implemented in daily neonatal work, making it easier to follow all changes after birth and to detect and discover very early the signs and symptoms that may evolve sooner or later during postnatal life. Foetal ultrasound can enable the discovery of major or minor congenital defects early in prenatal life, which helps perinatologists and parents to decide how to proceed with the pregnancy and to be prepared for known causes of problems in extrauterine life, such as congenital heart defects, diaphragmatic hernia, gastroschisis, and many other congenital malformations of greater or lesser importance. Cardiotocography is also now available in every delivery room to track foetal conditions.

The modern era of neonatal intensive care started in 1960, when the first neonatal intensive care unit was established by Professor Louis Gluck at Yale University in the United States [1]. Many predecessors deserve credit for the development of neonatal care all over the world, although the majority of them were situated in Western countries. The evolution of neonatology, as a new discipline, was well presented in an article written by Professor Alistair G.S. Philip [2]. I remember 1980 very well, because not only did I graduate from the Faculty of Medicine of the University of Ljubljana, Slovenia, but also because Fujiwara et al. finally succeeded with the application of artificial surfactant in ten premature infants with severe respiratory distress syndrome, eight of whom survived [3]. This was a major breakthrough, more than two decades after Marry Ellen Avery and Jere Maede, in the late 1950s, linked respiratory distress syndrome (RDS) with a deficiency of surfactant in the lung fluid, and it gave hope of survival and a future to thousands of premature infants with RDS [4].

Different modes of neonatal respiratory support have been developing concomitantly with other improvements in technological equipment, diagnostics, and the availability of new medicines, from the simplest respiratory apparatus to the most sophisticated, which have allowed different respiratory support modalities: different continuous positive airway pressures, CPAP, systems, and conventional and nonconventional respiratory support, such as high-frequency oscillatory ventilation [5,6]. Furthermore, noninvasive respiratory support has become preferable to its invasive counterpart because it involves less trauma and stress for the infant [7]. In extremely premature infants, a less invasive application of artificial natural surfactant has become the treatment of choice [8].

In the delivery room, a quick assessment of the readiness of a newborn for extrauterine life has been a cornerstone of preventing hypoxic–ischaemic events, regardless of their cause. Virginia Apgar published a score in 1953, which later became the famous APGAR score, of five vital signs that should be checked in each newborn at one and five minutes after birth, and resuscitation measures began to be started in the delivery room if the APGAR score was very low, showing nonreadiness for extrauterine life without the help of attending healthcare professionals [9]. However, as was concluded by Ehrenstein in her review and in the retrospective study among Danish conscripts, the associations of low Apgar scores with neurological disability have been shown with sufficient consistency, but the low associated absolute risks do not warrant the use of a low Apgar score to predict long-term neurologic prospects for individual infants [10,11].

With the further development of intensive neonatal care, smaller and smaller premature infants have been able to survive, now even those at 23 weeks of gestation and above. For a better outcome in these extremely low-gestational-age infants, specialized and regional tertiary level NICUs have been opened, together with possibilities of maternal transport to such tertiary regional centres if the delivery started prematurely or congenital anomalies mean that a further multidisciplinary team will be needed [12]. Such maternal transport or *transport* in utero *(TIU)* has been much better for the smallest infants, preventing them from suffering transport stress, hypothermia, difficulties in the management of cardiorespiratory support during retrieval from the referral maternity hospital, and so on. In 1984, the College of the Division of Gynaecology and Obstetrics at the University Medical Centre Ljubljana, Slovenia adopted a decision to introduce *TIU* in Slovenia. The first pregnant women were transferred in 1985 [13]. Better prenatal care has also involved the treatment of pregnant women with steroids as lung maturational therapy for foetal lungs if a premature delivery is expected (below 32 weeks of gestation [14]), antibiotics if infection is suspected, and MgSO_4_ if premature contractures have started, because MgSO_4_ also stabilizes the brain vascular circuitry in the most extremely premature infants. Analysis has shown a marked decrease in cerebral palsy and the combined risk of foetal/infant death and cerebral palsy at 2 years [15].

The most beneficial life support measure has been extracorporeal membrane oxygenation, ECMO, first started by R. Bartlett in the 1970s and later spreading all over the world for neonates and children when mechanical ventilation with all cardiocirculatory and inhalational support (i.e., inhalation of nitric oxide gas) has failed [16,17]. At the Neonatal and Paediatric Intensive Care Unit of the University Medical Centre Ljubljana, Slovenia, a Bartlett program was introduced after animal experiments into neonates and children who had suffered acute respiratory failure [18].

Some therapies, despite having been in use for many years, are still not optimal for the best recovery from hypoxic–ischaemic (HI) events during the perinatal period. One of these therapies is induced therapeutic hypothermia in neonates after HI insults that proceed to HI encephalopathy. The main issues that still need to be resolved or have not been resolved completely are: What is the evolution of injury after HI insult? What possible therapies, if any at all, can prevent secondary energy failure? Are therapeutic and neuroprotective hypothermia safe, alone or in combination with additional treatment options? The most recent meta-analysis concluded in 2021 that therapeutic hypothermia reduces the risk of death in neonates with moderate-to-severe hypoxic–ischaemic encephalopathy. Both selective head cooling and whole-body cooling methods are effective in reducing the mortality of infants with this condition. Moreover, low-income countries benefit the most from this therapy [19]. The sooner it is started, the better, so even during transportation to the tertiary level NICU, neonates should and can be effectively cooled [20].

The follow-up of children after NICU treatment is important because the results can be used to compare our work with international NICUs and to audit our work in areas that stand out in a negative direction. Vermont Oxford Network (VON) is one of the biggest networks in the world, in which more than 1200 NICUs participate to process their neonatal data [21]. The Slovenian NICU from the Maternity Hospital Division of Gynaecology and Obstetrics, University Medical Centre Ljubljana is one of these. A comparison of the care of premature babies with very low birth weight with data from the entire VON for 2021 showed that the results of our work in most of the analysed factors are good or even better than the VON average. The deviations found both in a worse average compared to other centres and in the deterioration of our results over several years (e.g., low body temperature in the first hour after admission to EINT, more frequent chronic lung disease, and more frequent severe retinopathy due to prematurity) can be the basis for introducing measures to improve results [22].

Last but not least, the ethical issues that arise in the care of critically ill infants are important. There are certainly many of them, but to mention only three: How should we approach extremely premature infants at the limit of viability? In addition, when and how to decide when faced with the end of life of critically ill infants and, lastly, the issue of interventional clinical trials with critically ill infants [23,24,25]. Space limitations do not allow these issues to be treated in detail, but they certainly should be discussed within teams. All decisions should be agreed and written with consensus [26].

There is still a lot of controversy and remaining work for the better prevention and treatment of common problems, especially in very and extremely premature babies, i.e., intraventricular haemorrhage, periventricular leukomalacia, retinopathy, respiratory distress syndrome, bronchopulmonary disease, necrotizing enterocolitis, sepsis, anaemia, osteopenia, metabolic derangements, growth problems, etc. Each of these problems would require a separate review and editorial. Enormous efforts by perinatologists, obstetricians, and neonatologists have been accomplished to prevent preterm delivery and treat any of those problems that arise. Despite all these efforts, extremely premature infants still suffer neurodevelopmental and cognitive problems, which may require long-term-life parental and social support [27,28].

Therefore, I am honoured to be the Guest Editor for this Special Issue dedicated to infants born at term or preterm who, because of their critical illness, need neonatal intensive care. I hope that the articles and research presented in this issue succeed in advancing the care of critically ill preterm and term infants. I am grateful to the authors and their teams for contributing to this Special Issue of *Children*, since the inspiring topics covered in this issue highlight the ongoing interest in promoting the diagnostics and treatment of term and premature infants in neonatal intensive care units. In the past and in the present, ongoing research into the wellbeing of foetuses and newborn infants and preventing them from becoming critically ill, as well the early recognition of a critically ill infant’s needs, contribute to quick diagnostics and treatment for a better outcome, with no or low risk of an adverse one.
